# Comparative Analysis of Methanogenic Communities in Different Laboratory-Scale Anaerobic Digesters

**DOI:** 10.1155/2016/3401272

**Published:** 2016-12-15

**Authors:** Ayrat M. Ziganshin, Elvira E. Ziganshina, Sabine Kleinsteuber, Marcell Nikolausz

**Affiliations:** ^1^Institute of Fundamental Medicine and Biology, Kazan (Volga Region) Federal University, Kazan, Republic of Tatarstan 420008, Russia; ^2^Department of Environmental Microbiology, Helmholtz Centre for Environmental Research-UFZ, 04318 Leipzig, Germany

## Abstract

Comparative analysis of methanogenic archaea compositions and dynamics in 11 laboratory-scale continuous stirred tank reactors fed with different agricultural materials (chicken manure, cattle manure, maize straw, maize silage, distillers grains, and* Jatropha* press cake) was carried out by analysis of the methyl coenzyme-M reductase *α*-subunit (*mcrA*) gene. Various taxa within Methanomicrobiales, Methanobacteriaceae, Methanosarcinaceae, Methanosaetaceae, and Methanomassiliicoccales were detected in the biogas reactors but in different proportions depending on the substrate type utilized as well as various process parameters. Improved coverage and higher taxonomic resolution of methanogens were obtained compared to a previous 16S rRNA gene based study of the same reactors. Some members of the genus* Methanoculleus *positively correlated with the relative methane content, whereas opposite correlations were found for* Methanobacterium*. Specific biogas production was found to be significantly correlating with Methanosarcinaceae. Statistical analysis also disclosed that some members of the genus* Methanoculleus *positively correlated with the ammonia level, whereas the prevalence of* Methanocorpusculum*,* Methanobacterium*, and* Methanosaeta* was negatively correlated with this parameter. These results suggest that the application of methanogenic archaea adapted to specific feedstock might enhance the anaerobic digestion of such waste materials in full-scale biogas reactors.

## 1. Introduction

Anaerobic digestion (AD) of organic matter is widely used in the treatment of agricultural and food wastes as well as industrial and domestic wastewaters. Moreover, the production of biogas from renewable biomass is a substantial way to partially shift from fossil fuels to renewable greenhouse gas-neutral bioenergy in order to mitigate the climate change. Currently, AD is intensively applied for the generation of clean energy and high-quality organic fertilizers from various organic substrates in many countries [1–4].

The AD process is driven by the activity of complex microbial consortia comprising different functional groups of bacteria and archaea that convert high molecular weight organic compounds into energy-rich methane in the absence of exogenous electron acceptors. The conversion of organic matter into biogas includes four sequential stages: hydrolysis, acidogenesis, acetogenesis, and methanogenesis. The bacterial community converts organic compounds to organic acids, alcohols, hydrogen, and carbon dioxide, whereas methanogenic members of the domain Archaea produce methane using acetoclastic, hydrogenotrophic, and methylotrophic pathways. The syntrophic relationship between bacteria oxidizing organic acids and alcohols and methanogenic archaea is essential for the AD process. Among various microorganisms involved in biogas generation, methanogens are very sensitive to different environmental factors, such as high ammonia, sulfide, and organic acids concentrations, leading to process impairments. Therefore, a detailed understanding of the microbial consortia in biogas reactors is important to fundamentally and practically develop and improve anaerobic digestion processes [5–8].

Methanogenic archaea involved in various AD processes include the strict hydrogenotrophic orders Methanomicrobiales, Methanobacteriales, and Methanococcales (with the predominance of distinct members of Methanomicrobiales and Methanobacteriales) as well as acetate-utilizing representatives of the order Methanosarcinales (with the predominance of members of the two genera* Methanosarcina* and* Methanosaeta*). Unlike* Methanosaeta* species, most* Methanosarcina* species can additionally use hydrogenotrophic and methylotrophic pathways of methanogenesis [9, 10].* Methanosarcina* species are able to achieve stable growth at high organic loading rates (OLR) and high levels of ammonium and acetate. In contrast, high ammonium concentrations and elevated acetate levels were reported to suppress the growth of* Methanosaeta* species [6, 11, 12]. However, the presence of some ammonia tolerant Methanosaetaceae-related microorganisms has also been observed [13, 14]. Furthermore, an increased activity of* Methanosaeta* spp. was discovered during shortening the hydraulic retention time (HRT) despite the high levels of organic acids, a result which was additionally supported by stable isotope fingerprinting of the biogas [15, 16]. Different hydrogenotrophic methanogens can form close associations with syntrophic acetate-oxidizing bacteria (SAOB; e.g.,* Clostridium ultunense* [17],* Syntrophaceticus schinkii* [18], and* Tepidanaerobacter acetatoxydans* [19]) at elevated temperatures or high ammonia levels. However, the diversity of such syntrophic bacteria and such interactions between bacteria and archaea should be further investigated. In addition, another order of methanogens, Methanomassiliicoccales, producing methane by reducing methanol with hydrogen as the electron donor, has been discovered in various anaerobic environments and became a new object for recent investigations [20, 21]. Therefore, the knowledge about the archaeal consortia in diverse anaerobic reactor systems is of practical interest in order to comprehensively understand and control the AD process, mitigate process disturbances, and maximize the methane yield.

In a previous study [22], we investigated the effects of substrate type and various process parameters on the bacterial and archaeal community structure using 16S rRNA gene analysis in 11 different reactor systems. However, the rRNA approach applied in our previous work does not completely reflect the actual community composition of methanogenic archaea due to its lower phylogenetic resolution [15, 16, 23]. Moreover, the relative abundance data obtained by 16S rRNA gene analysis is biased due to the different copy numbers of rRNA operons in various methanogenic taxa (e.g.,* Methanoculleus*: 1 copy;* Methanosaeta*: 1-2 copies;* Methanococcus*: 2–4 copies;* Methanosarcina*: 3 copies;* Methanospirillum*: 4 copies according to https://rrndb.umms.med.umich.edu/) [24], while* mcrA* is a single-copy gene in most methanogens (in rare cases, an additional* mrt* gene encoding an isoenzyme is found in members of Methanobacteriales and Methanococcales). Mcr catalyzes the final step of methanogenesis and is found in the genomes of all methanogenic archaea [25–27].

More detailed knowledge on the relations between various reactor parameters (e.g., temperature, pH, OLR, and HRT as well as ammonia and organic acids levels) and the ecophysiology of methanogens involved in AD can help develop new effective tools to stabilize methanogenesis in biogas reactors and improve their efficiency. Thus, the main goal of this study which continues our previous investigations was to estimate the influence of different process parameters on the methanogenic communities involved in the AD of various agricultural materials (chicken manure, cattle manure, maize straw, maize silage, distillers grains, and* Jatropha* press cake). The composition and dynamics of the methanogenic communities were investigated in laboratory-scale continuous stirred tank reactors (CSTR) targeting the* mcrA *genes and comparing the results with the archaeal 16S rRNA gene data obtained previously. Correlations between the abiotic process parameters and the structure of the methanogenic communities were additionally investigated.

## 2. Materials and Methods

### 2.1. Laboratory-Scale Biogas Reactors and Analytical Techniques

The experiment was started after all reactors had been running under stable conditions for at least threefold HRT to ensure steady state conditions. Table S1 (Supporting Information, SI, available online at http://dx.doi.org/10.1155/2016/3401272) shows the main process parameters measured at three sampling times during the operating period of the reactors (for more details, see Ziganshin et al. [22]). Briefly, 11 laboratory-scale reactors were operated under mesophilic conditions (37–40°C) except that reactors R4.5 and R4.6 were changed to thermophilic conditions (55°C) between the second and the third sampling points. After the second sampling point, both R4.5 and R4.6 were operated at 39°C for one week, and then the operation temperature was changed to 55°C at 0.9–1.1°C/day. Before the third sampling point, both reactors were operated at 55°C for 6 days. Reactor R3.1 with a working volume of 36.5 L was fed with chicken manure and cattle manure and had a specific biogas potential (SBP) of 290–390 mL g^−1^_VS_ depending on the OLR. Reactor R4.5 with a 12–8 L working volume was set up only with cattle manure (SBP: 250–590 mL g^−1^_VS_ depending on OLR and temperature), whereas reactor R4.6 with a working volume of 8 L was set up with cattle manure and dried distillers grains with solubles (DDGS) (SBP: 520–540 mL g^−1^_VS_ depending on OLR and temperature). Reactor R4.8 with 10 L working volume (SBP: 340–380 mL g^−1^_VS_) and reactor R4.17 with a working volume of 100 L (SBP: 590–720 mL g^−1^_VS_ depending on OLR) were fed with cattle manure and maize silage. The feedstock for reactors R4.13 and R4.14 with 30 L working volume was composed of cattle manure and maize straw (SBP: 330–400 mL g^−1^_VS_ depending on OLR); the feedstock for reactors R4.15 and R4.16 with the same volume consisted of cattle manure and extruded maize straw (SBP: 380–410 mL g^−1^_VS_ depending on OLR). Reactors R4.19 and R4.20 which had a working volume of 9 L treated* Jatropha* press cake (SBP: 450–490 mL g^−1^_VS_ depending on OLR). Biogas volume, methane content, and pH values were measured every day whereas acid capacity, concentration of volatile fatty acids (VFA), and total ammonium nitrogen (TAN) concentrations were measured twice per week as previously described (Table S1) [22]. Samples for community analysis were taken on day 1, day 35, and day 63 of reactor operation.

### 2.2. DNA Extraction and PCR Amplification of* mcrA*

DNA extraction and purification were performed using the FastDNA SPIN Kit for soil (MP Biomedicals). DNA was checked for integrity by agarose gel electrophoresis and quantified with a NanoDrop ND-1000 UV-Vis spectrophotometer (Thermo Fisher Scientific). Amplification of the* mcrA* genes was carried out with the primers mlas (5′-GGT GGT GTM GGD TTC ACM CAR TA-3′) and mcrA-rev (5′-CGT TCA TBG CGT AGT TVG GRT AGT-3′) using the PCR protocol described previously [16].

### 2.3. T-RFLP Analysis

For terminal restriction fragment length polymorphism (T-RFLP) analysis, the reverse primer was labeled at the 5′-end with phosphoramidite fluorochrome-5-carboxyfluorescein. Fluorescently labeled amplicons were purified with SureClean Plus (Bioline) and digested with* Hae*III and* Msp*I restriction enzymes (New England Biolabs) in separate reactions. GeneScan-500 ROX (Applied Biosystems) standard was used to obtain molecular sizing of the terminal restriction fragments (T-RFs). Fluorescently labeled T-RFs were sized on an ABI PRISM 3130xl Genetic Analyzer (Applied Biosystems), and peaks < 50 bp and > 500 bp were removed from the subsequent analysis. Theoretical T-RF values of the* mcrA* amplicons were calculated with NEBcutter V2.0 (http://tools.neb.com/NEBcutter2/) and confirmed experimentally by T-RFLP analysis of corresponding clones. T-RFLP analysis was conducted in triplicate for each restriction analysis to ensure reproducibility. An R script (R version 2.12.2; http://www.r-project.org/) with a cut-off value of six times the standard deviation was used to remove background noise [28]. Multivariate statistical analysis using the vegan package of R (version 3.0.1) was performed on the T-RFLP profiles applying the Bray-Curtis dissimilarity index [29] as described previously [16, 22]. Correlations between the abundance of different T-RF and various reactor parameters were analyzed with the R Hmisc package (based on Spearman's rank correlation coefficient).

### 2.4. Cloning and Sequencing

PCR products were purified with the QIAGEN PCR Purification Kit, and cloning was performed using the QIAGEN PCR Cloning Kit. For each selected reactor, about 50 to 100* mcrA* gene positive clones were chosen and screened by T-RFLP analysis to find inserts matching the dominant peaks of the community T-RFLP patterns. Selected clones from each group were sequenced on an ABI PRISM 3130xl Genetic Analyzer. Data were checked for chimeric sequences with Bellerophon (http://comp-bio.anu.edu.au/bellerophon/bellerophon.pl) [30]. Sequences were compared to public databases using BLASTX and BLASTN programs (http://www.ncbi.nlm.nih.gov/BLAST) excluding environmental clone sequences. Phylogenetic trees were calculated with MEGA5 using the neighbor-joining method based on Jukes-Cantor evolutionary distances [31]. The partial* mcrA* sequences obtained in this study were deposited in the GenBank database (accession numbers KX523626–KX523674).

## 3. Results

### 3.1. Methanogenic Community Composition Based on* mcrA* Gene Analysis

**Figure 1:**
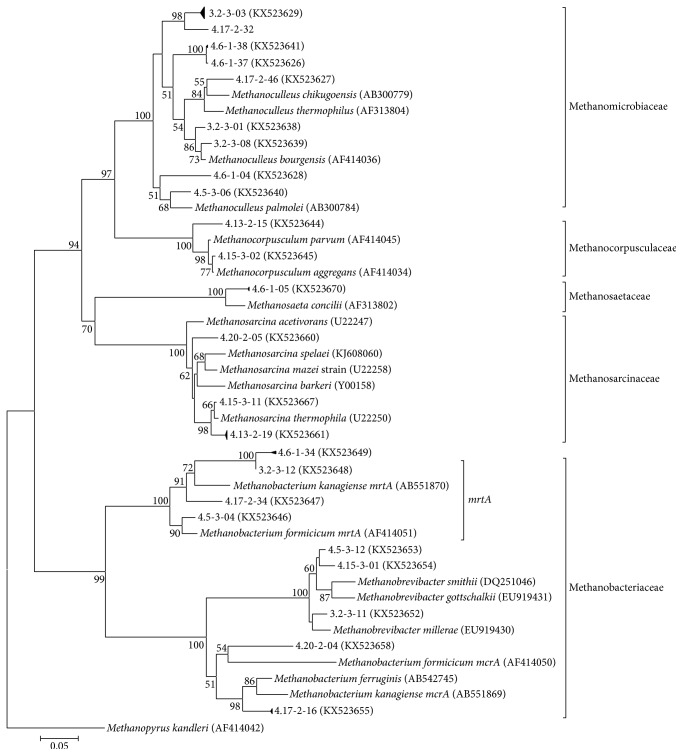
Phylogenetic tree indicating the relationship of selected* mcrA* and* mrtA* gene sequences to those retrieved from methanogenic strains. Analysis was conducted in MEGA5 using the neighbor-joining method based on Jukes-Cantor evolutionary distances. The percentages of replicate trees in which the associated taxa clustered together in the bootstrap test (1000 replicates) are shown next to the branches. Branches containing more closely related clone sequences were compressed and only one selected clone is shown. Accession numbers of the sequences are shown in brackets.* Methanopyrus kandleri* was used as outgroup reference. The scale bar represents 5% sequence divergence.

**Figure 2:**
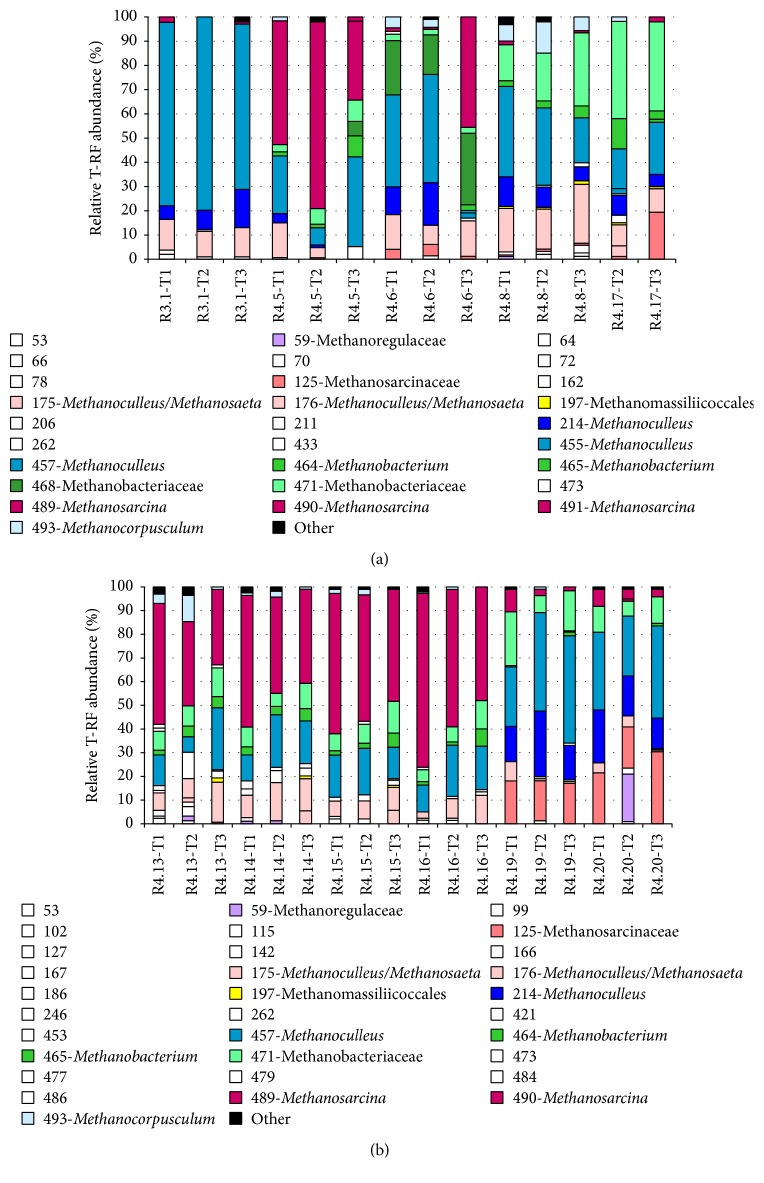
Community structure and dynamics of methanogenic archaea in 11 reactors at three sampling times (T1, T2, and T3; see Table S1 for details) according to T-RFLP profiles of* mcrA *amplicons digested with the restriction enzyme* Hae*III (reactors R3.1, R4.5, R4.6, R4.8, and R4.17 (a); reactors R4.13, R4.14, R4.15, R4.16, R4.19, and R4.20 (b)). Only T-RFs comprising at least 1% relative abundance in at least one sample are presented. Laboratory-scale reactors were operated under mesophilic conditions (37–40°C) except that reactors R4.5 and R4.6 were changed to thermophilic conditions (55°C) between the second and the third sampling points.* Methanoculleus* and* Methanosaeta* members had very similar T-RF sizes (176 and 175, resp.; Table S2, SI) after digestion of* mcrA* amplicons with* Hae*III. Thus, their differentiation in R3.1 was additionally achieved by using* Msp*I analysis in a separate reaction, which indicated the absence of strict acetoclastic methanogens in this reactor. In other reactors, the proportion of both phylotypes varied. R3.1 was fed with chicken manure and cattle manure; R4.5 was set up only with cattle manure; R4.6 was set up with cattle manure and DDGS; R4.8 and R4.17 were fed with cattle manure and maize silage; R4.13 and R4.14 were fed with cattle manure and maize straw; R4.15 and R4.16 were set up with cattle manure and extruded maize straw; R4.19 and R4.20 treated* Jatropha* press cake.

Methanogenic archaeal community dynamics in reactors were tracked by T-RFLP fingerprinting analysis combined with clone sequencing as a cost-effective method for the identification of methanogenic communities [32]. The highest BLASTX hits of representative* mcrA* gene clones and their T-RF values obtained with* Hae*III and* Msp*I enzymes are summarized in Table S2 (Supplementary Material). The neighbor-joining tree constructed from* mcrA* nucleotide sequences is shown in Figure 1. The majority of* mcrA* sequences were closely related to clones from various anaerobic digesters. In general, a good agreement was obtained with both the* mcrA* and the archaeal 16S rRNA analyses, but the rRNA gene approach missed the representatives of the family Methanobacteriaceae. According to BLASTX hits, various operational taxonomic units (OTUs) were affiliated with the hydrogenotrophic order Methanomicrobiales (*Methanoculleus* spp.,* Methanocorpusculum* sp., and unclassified Methanoregulaceae), the hydrogenotrophic family Methanobacteriaceae (*Methanobacterium* spp. and* Methanobrevibacter* spp.), the acetoclastic/methylotrophic/hydrogenotrophic Methanosarcinaceae (*Methanosarcina* spp. and* Methanomethylovorans* sp.), the acetoclastic Methanosaetaceae (*Methanosaeta* sp.), and the hydrogen-dependent methylotrophic Methanomassiliicoccales order.  Figure 2 shows the T-RFLP patterns generated after digestion of* mcrA* amplicons with* Hae*III. Similar results were obtained with* Msp*I (data not shown).

No significant differences were observed in the community structure of reactor R3.1 fed with chicken and cattle manure, when data for both* mcrA *and 16S rRNA genes were analyzed and compared. Methanogenic community based on the* mcrA *gene profiles in R3.1 was dominated by members of the hydrogenotrophic* Methanoculleus* genus (94–98% of T-RF abundance in all samples) and was presented by T-RF 176, T-RF 214, and T-RF 455/457 (Figure 2(a)).* Methanoculleus* with T-RF 176 (OTU 2 in the clone library) showed 86–91%* mcrA* gene sequence similarity with other* Methanoculleus* phylotypes detected in this study, while* Methanoculleus* with T-RF 214 (OTU 3 in the clone library) showed 97-98% and 88–93%* mcrA* gene sequence similarity with OTUs 4 and 5 (*Methanoculleus* with T-RF 455/457), respectively.* Methanoculleus* and* Methanosaeta* members had very similar T-RF sizes (176 and 175, resp.; Table S2, SI) after digestion of* mcrA* amplicons with* Hae*III. Thus, their differentiation in R3.1 was additionally achieved by using* Msp*I analysis in a separate reaction, which indicated the absence of strict acetoclastic methanogens in this reactor. Most of the sequenced* mcrA* gene clones belonging to* Methanoculleus* had high similarities to uncultured methanogens and shared 93–100% BLASTX identity with* mcrA* sequences of* Methanoculleus bourgensis* [33],* Methanoculleus palmolei* [34], and* Methanoculleus chikugoensis* [35] strains (Table S2, SI).* Methanoculleus *with T-RF 455/457 was also found in high proportions in all other reactors (Figure 2). T-RF 214, also assigned to the* Methanoculleus* genus, was detected at notable abundance in reactors fed with* Jatropha* press cake, cattle manure, and DDGS (mesophilic conditions) as well as cattle manure and maize silage under various operating conditions (Figure 2).* Methanoculleus *phylotypes were also found in all reactors when the rRNA gene approach was applied [22]; however, their proportions in the samples varied.

The next predominant taxon was the family Methanobacteriaceae with T-RF 464/465, T-RF 468, and T-RF 471. Members of this group were found in all reactors with the exception of R3.1 that operated at high ammonia and VFA levels. The rRNA gene approach missed the family Methanobacteriaceae, whereas analysis of* mcrA* genes allowed distinguishing the genera* Methanobacterium* and* Methanobrevibacter* within the family Methanobacteriaceae (Figure 2). The* mcrA* sequence type with T-RF 471 had 94–97% BLASTX identity to* mcrA* of* Methanobacterium formicicum* [36],* Methanobacterium kanagiense* [37],* Methanobrevibacter smithii* [38], and* Methanobrevibacter gottschalkii* [39] (Table S2, SI). Methanobacteriaceae with T-RF 468 were mostly observed in reactor R4.6 fed with cattle manure and DDGS during the whole experimental period (T-RF abundance of 16–30%), whereas sequences affiliated to Methanobacteriaceae with T-RF 471 were notably detected in reactors fed with cattle manure and maize silage (R4.8 and R4.17; 15–40% T-RF abundance) and* Jatropha* press cake (R4.19 and R4.20; 7–23% T-RF abundance) as well as in reactors fed with cattle manure and maize straw (R4.13–R4.16) but in lower proportions (T-RF abundance of 5–13%; Figure 2).

The next major group identified within the T-RFLP profiles of most reactors was the genus* Methanosarcina* with T-RF 125 and T-RF 489/491.* Methanosarcina* with T-RF 125 showed 91-92%* mcrA* gene sequence similarity with* mcrA* gene of* Methanosarcina* with T-RF 489/491. In addition, these* mcrA* genes shared 94–97% BLASTX identity with* mcrA* of* Methanosarcina acetivorans* [40],* Methanosarcina thermophila* [41], and* Methanosarcina spelaei* [42] strains (Table S2, SI).* Methanosarcina* with T-RF 489/491 was identified as an important group in reactors R4.13 and R4.14 fed with cattle manure and maize straw. Furthermore, the same phylotype dominated in reactors R4.15 and R4.16 fed with cattle manure and extruded maize straw. However, the relative abundance of* Methanosarcina* decreased during the operation of these reactors (from 51–56% to 32–40% T-RF abundance in R4.13 and R4.14 and from 59–73% to 47-48% T-RF abundance in R4.15 and R4.16) (Figure 2(b)). A similar trend had been observed from the same samples by using the rRNA gene approach [22], but in different proportions.

T-RF 489/491 (*Methanosarcina* sp.) was also predominant in reactor R4.5 fed with cattle manure and its relative abundance reached 77% in the second sample (Figure 2(a)). After the second sampling, the temperature in reactor R4.5 was gradually increased to a maximum of 55°C. This temperature shift was accompanied by a decrease of* Methanosarcina* sp. to about 34% T-RF abundance and by an increase of* Methanoculleus *sp. (T-RF 455/457) from 7% up to 37% T-RF abundance. A similar trend had been observed from the same samples with the rRNA gene approach [22]. In case of reactor R4.6 fed with cattle manure and DDGS, the temperature shift to 55°C contrarily resulted in strong inhibition of* Methanoculleus* with T-RF 214 and T-RF 455/457 and in the appearance of* Methanosarcina* with T-RF 489/491 (from 2% to 46% T-RF abundance). In addition, another* Methanosarcina* with T-RF 125 was mostly observed in all samples from reactors fed with* Jatropha* press cake (with T-RF abundance of 17–30%), but* Methanosarcina* with T-RF 489/491 was detected at low T-RF abundance of 2–9% in these reactors (Figure 2(b)).

Based on the* mcrA* gene analysis, in contrast to the rRNA gene data [22], lower proportions of* Methanosaeta* (T-RF 175) were observed in the first and second samples from reactor R4.6. OTU with T-RF 175 had 95-96% BLASTX identity to* mcrA* of* Methanosaeta concilii* [43].* Methanosaeta* representatives were also detected in maize silage-fed reactors (Figure 2) but in much lower proportions compared to the results using the rRNA gene approach [22]. Other archaea of the genera* Methanocorpusculum* (T-RF 493) and* Methanomethylovorans* (T-RF 124) were detected in some samples but in low proportions (Figure 2). OTU with T-RF 493 had 95–99% BLASTX identity with* mcrA* of* Methanocorpusculum aggregans* [44] (Table S2, SI). OTU with T-RF 124 had 96% BLASTX identity with* mcrA* of* Methanomethylovorans thermophila* [45] (Table S2, SI).

### 3.2. Correlations between Methanogenic Communities and Abiotic Process Parameters

**Figure 3:**
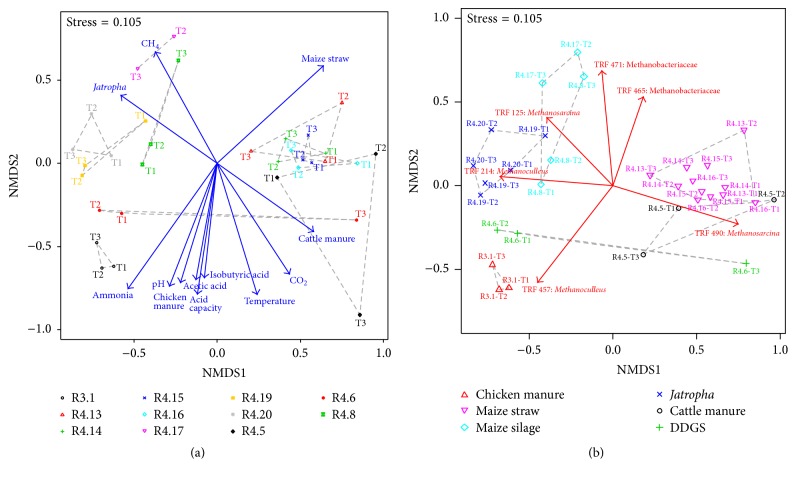
Nonmetric multidimensional scaling plots of T-RFLP profiles of* mcrA* amplicons digested with the restriction enzyme* Hae*III. (a) Blue arrows indicate the vectors of process parameters which shaped community differences the most (significance factors *p* < 0.01, tested by Monte-Carlo permutation against 1000 random data sets). (b) Samples from reactors fed with the same substrate are marked with the same color. Red arrows indicate vectors of single T-RF (significance factors 0.01 < *p* < 0.05) which shaped the communities the most.

Figure 3 illustrates the results of a multivariate statistical analysis shown in nonmetric multidimensional scaling (NMDS) plots calculated from the T-RFLP profiles of* mcrA* amplicons digested with* Hae*III.  Figure 3(a) demonstrates very distinct community structures in most reactors, and almost all samples from the same reactor clustered more closely to each other with the exception of the two samples from R4.5 and R4.6 taken at higher temperature (55°C) and samples from R4.8 which were relatively scattered within the NMDS plot. Vectors of process parameters show that the most decisive abiotic factors shaping the methanogenic community structure in reactor R3.1 were high pH and high TAN and VFA concentrations (acetic and isobutyric acids). The community shift after the temperature change from 38°C to 55°C was reflected by the data points of the samples obtained from R4.5 and R4.6 as well (Figure 3(a)). In addition, Figure 3(b) shows the NMDS plot demonstrating vectors of single T-RF which shaped the community composition the most.

**Figure 4:**
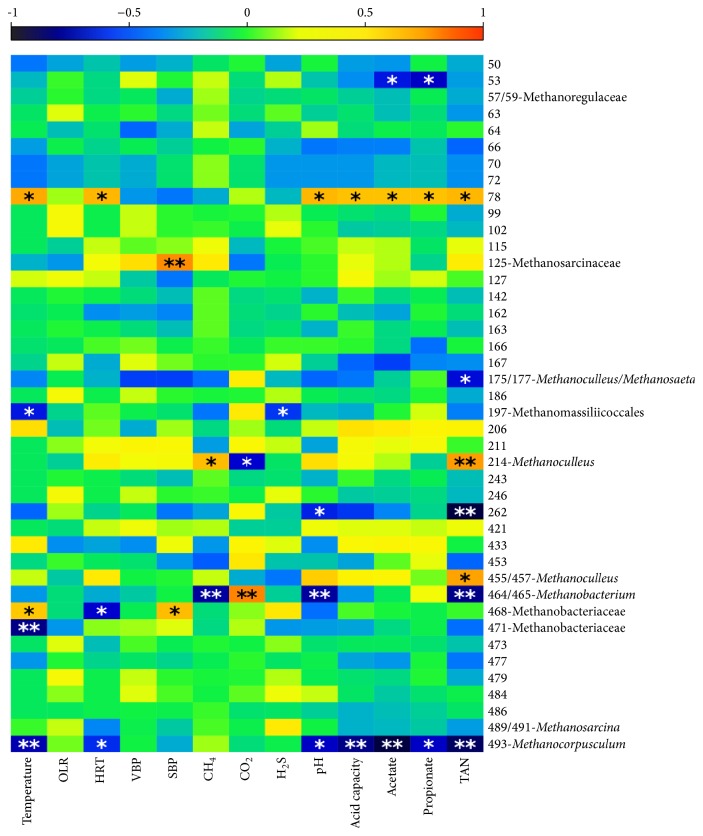
Correlations between the abundance of different taxa and various process parameters in biogas reactors' samples. Spearman's correlation coefficients are shown by color ranging. Negative correlations are displayed in blue color while positive correlations are displayed in red color. Significant correlations are indicated by ^*∗*^ *p* < 0.01 and ^*∗∗*^ *p* < 0.001.

The relationships between archaeal community members and various abiotic process parameters were investigated additionally by correlation analysis (Figure 4). The applied analysis revealed that relative abundance of several OTUs correlated with different process parameters. Thus, the abundance of Methanomassiliicoccales (T-RF 197), Methanobacteriaceae (T-RF 471), and* Methanocorpusculum* (T-RF 493) was negatively correlated with the reactor temperature (*ρ* = −0.49, −0.63, and −0.56, resp.), whereas the abundance of Methanobacteriaceae (T-RF 468) was positively correlated with the temperature (*ρ* = 0.47). A significant correlation of SBP with the abundance of Methanosarcinaceae (T-RF 125) and Methanobacteriaceae (T-RF 468) (*ρ* = 0.60 and 0.50, resp.) was also found. The abundance of* Methanoculleus *(T-RF 214) was positively correlated with the methane content (*ρ* = 0.49) and was negatively correlated with the carbon dioxide content (*ρ* = −0.52). In contrast, opposite correlations were found for* Methanobacterium* (T-RF 464/465) that negatively correlated with the methane content (*ρ* = −0.59) and positively correlated with the carbon dioxide content (*ρ* = 0.62). The abundance of* Methanobacterium* (T-RF 464/465) and* Methanocorpusculum* (T-RF 493) was negatively correlated with the pH value (*ρ* = −0.60 and −0.53, resp.). The abundance of* Methanocorpusculum* sp. was additionally negatively correlated with the acid capacity and acetate, propionate, and TAN concentrations (*ρ* = −0.62, −0.75, −0.54, and −0.70, resp.). Moreover, TAN concentration was found to be a significant factor for* Methanoculleus *(T-RF 214 and T-RFs 455/457), which positively correlated with this parameter (*ρ* = 0.57 and 0.55, resp.), and also for* Methanobacterium* (T-RF 464/465; *ρ* = −0.62) and* Methanosaeta* (T-RF 175), which negatively correlated with the TAN level.

## 4. Discussion

Despite the fact that comparable results were obtained using both approaches,* mcrA* data additionally allowed the identification of various members of the family Methanobacteriaceae which were missed with the applied 16S rRNA gene-specific primer set in the previous study. Moreover, the relative abundance data based on* mcrA* gene were less biased compared to the rRNA gene based approach which is impacted by the gene copy number variability.

Based on the analysis of* mcrA* as well as 16S rRNA genes, the methanogenic community in reactor R3.1 fed with chicken and cattle manure was less diverse compared to the other ten reactors and was dominated by members of the genus* Methanoculleus*, indicating strong inhibition of the acetoclastic pathway of methanogenesis. The closely related strains are hydrogenotrophic methanogens that can utilize H_2_/CO_2_ or formate as methanogenic substrates. The most obvious explanation for the inhibition of acetoclastic methanogenesis in R3.1 (the frequency of members of Methanosarcinaceae never exceeded 1.5%) is the high concentration of TAN and free ammonia nitrogen (FAN) (up to 5.9 g L^−1^ and 0.74 g L^−1^, resp.) accumulated during the reactor operation at increasing OLR (up to 2.84 g_VS_ L^−1^day^−1^; Table S1, SI). However, the prevalence of* Methanosarcina* in reactors utilizing chicken manure as the sole substrate and operating at high TAN/FAN levels was also reported [46, 47], indicating that substrate differences and the inoculum used to start the process might have a crucial impact on the development of microorganisms. In addition, reactor R3.1 operated at high levels of VFA (primarily of acetate and propionate, up to 9.9 g L^−1^ and 4.1 g L^−1^, accordingly). Therefore, instead of acetoclastic methanogenesis, an alternative acetate sink via the activity of syntrophic acetate-oxidizing bacteria (SAOB) possibly occurred in R3.1 as previously reported by Schnürer and Nordberg [48] and Fotidis et al. [49] in the presence of high amounts of ammonia (above 2.8–3 g L^−1^ ammonium nitrogen). The prevalence of members of the genus* Methanoculleus* in this reactor indicates their ability to dominate under extreme conditions (*Methanoculleus *phylotypes with T-RF 214 and T-RF 455/457 positively correlated with TAN and FAN levels) (Supplementary Information, Fig. S1). Representatives of the genus* Methanoculleus *were also found in all other reactors fed with distinct agricultural substrates indicating that they are the key players of hydrogenotrophic methanogenesis under various conditions. Other studies also identified the genus* Methanoculleus* as a widespread methanogen in various biogas reactors (e.g., [23, 50–54]).* Methanoculleus bourgensis *MS2^T^ strain sharing high sequence similarity with clone sequences from the T-RF 455/457 group was successfully applied in a previous bioaugmentation experiment to alleviate the ammonia toxicity effect [55]. A fivefold increase in relative abundance of* Methanoculleus* spp. and a 31% increase in methane yield were observed in the bioaugmented CSTR compared to the control, indicating that bioaugmentation can help to solve the toxicity problem associated with ammonia overload in biogas reactors operating with nitrogen-rich feedstock.

Members of the family Methanobacteriaceae were found at high levels in all reactors with the exception of reactor R3.1 fed with chicken and cattle manure, indicating the sensitivity of some species to high ammonia and VFA concentrations. The representatives of this family are strict hydrogenotrophic methanogens, growing and forming methane from either H_2_/CO_2_ or formate [36, 39]. The rRNA gene approach missed the family Methanobacteriaceae, whereas analysis of* mcrA* genes allowed distinguishing the genera* Methanobacterium* and* Methanobrevibacter* within Methanobacteriaceae. Interestingly,* Methanobacterium* with T-RF 464/465 negatively correlated with TAN level, pH, and CH_4_ content (*p* < 0.001), while Methanobacteriaceae with T-RF 471 only negatively correlated with temperature (*p* < 0.001). Members of the family Methanobacteriaceae were detected in different anaerobic digesters (e.g., [23, 46]) as well as other environments [56] and in parallel with Methanomicrobiales representatives (e.g.,* Methanoculleus*) they were reported to be involved in the SAO process at high ammonia levels (>2.8 g TAN L^−1^) [49]. Therefore, SAO can also be assumed in reactors R4.19 and R4.20 fed with* Jatropha* biomass which operated at elevated levels of TAN (3.2–3.8 g L^−1^; Table S1, SI).

The next group, members of which were found at high levels in all biogas reactor systems but in various proportions, was the genus* Methanosarcina*. The related strains are acetoclastic and methylotrophic methanogens and some can also utilize H_2_/CO_2_ as methanogenic substrates. Methanogens of the genus* Methanosarcina* (T-RF 489/491) were also discovered among T-RF profiles as dominant microorganisms in most samples of reactor R4.5 fed with cattle manure as well as in reactors R4.13, R4.14, R4.15, and R4.16 fed with cattle manure and maize straw.* Methanosarcina* with T-RF 489/491 started to dominate in reactor R4.6 fed with cattle manure and DDGS after the gradual temperature change from mesophilic to thermophilic conditions. All these reactors operated at medium levels of ammonia (in the range of 1.2–2.0 g L^−1^ and 0.05–0.14 g L^−1^ of TAN and FAN, resp.; Table S1, SI). Additionally, another* Methanosarcina* with T-RF 125 was mostly observed in all samples from reactors R4.19 and R4.20 fed with* Jatropha* press cake. Reactors R4.19 and R4.20 operated at higher TAN and FAN levels (up to 3.8 g L^−1^ and 0.29 g L^−1^, resp.) compared to all other reactors (with the exception of R3.1; Table S1, SI). This indicates that different methanogenic species even within the same genus can respond differently to ammonia concentrations.* Methanosarcina* species can perform acetoclastic methanogenesis and have been suggested to act as hydrogen-consuming microorganisms during SAO in reactors operating at high ammonia levels [12, 57].* Methanosarcina* spp. are found in various environments [56] and reported to achieve stable growth at high OLR and high levels of ammonium in biogas reactors, indicating their tolerance towards different process impairments [12], and therefore they are frequently found in various biogas reactors (e.g., [8, 14, 47, 55, 58]).

Other methanogenic archaea exclusively conducting acetoclastic methanogenesis,* Methanosaeta* spp. [43, 59], were also detected in some reactors in this study. TAN concentration was found to be a significant factor for* Methanosaeta*, the abundance of which was negatively correlated with this key parameter of AD. In addition, lower acetate values were found in reactors R4.6 and R4.8 operated under mesophilic conditions compared to other experiments. It was previously shown that reactors with low concentrations of ammonia and VFA were dominated by representatives of Methanosaetaceae, whereas reactors with high levels of ammonia and VFA were dominated by members of Methanosarcinaceae [6, 11].

## 5. Conclusions

This study investigated the effect of substrate type and various process parameters on composition and dynamics of methanogenic communities based on* mcrA* genes in 11 laboratory-scale biogas reactors operated with different agricultural waste materials. A good agreement was found with the 16S rRNA data obtained previously, but the rRNA gene approach missed the family Methanobacteriaceae while the* mcrA *gene approach allowed more detailed differentiation of methanogenic taxa. Furthermore, the relative abundance data obtained by the* mcrA* gene gives better results since the 16S rRNA gene data is more biased due to the different copy numbers of rRNA operons in various archaeal taxa. Multivariate statistics revealed that the decisive process parameters shaping the methanogenic community structure in the reactor fed with chicken manure were the high TAN level, pH, and VFA concentration. The present study shows that application of methanogenic communities in biogas reactors adapted to specific feedstock might improve the anaerobic digestion of such waste materials in full-scale biogas reactors.

## Supplementary Material

Table S1. Operating conditions and process parameters.
Table S2. Sequencing results of representative mcrA gene clones and related terminal restriction fragments (T-RF).
Fig. S1. Relationships between FAN concentration and abundance of Methanoculleus (T-RF 455/457).

## Abbreviations

AD: Anaerobic digestion
CSTR: Continuous stirred tank reactor
FAN: Free ammonia nitrogen
HRT: Hydraulic retention time
NMDS: Nonmetric multidimensional scaling
OLR: Organic loading rate
OTU: Operational taxonomic unit
SAO: Syntrophic acetate oxidation
SAOB: Syntrophic acetate-oxidizing bacteria
SBP: Specific biogas production
TAN: Total ammonia nitrogen
T-RF: Terminal restriction fragment
T-RFLP: Terminal restriction fragment length polymorphism
VBP: Volumetric biogas production
VFA: Volatile fatty acids
VS: Volatile solids.
