# Supplementary material for: Comparative Analysis of Methanogenic Communities in Different Laboratory-Scale Anaerobic Digesters
Source: Archaea. 2016 Dec 15;2016:3401272. doi: 10.1155/2016/3401272 (PMC5198152; doi:10.1155/2016/3401272)
Supplement: Supplementary file 1 — Table S1. Operating conditions and process parameters. Table S2. Sequencing results of representative mcrA gene clones and related terminal restriction fragments (T-RF). Fig. S1. Relationships between FAN concentration and abundance of Methanoculleus (T-RF 455/457). [file 3401272.f1.pdf]

Supplementary information to the article entitled:

Comparative Analysis of Methanogenic Archaeal Communities in Different laboratory Anaerobic Digesters

Authors are:

Ayrat M. Ziganshin, Elvira E. Ziganshina, Sabine Kleinsteuber and Marcell Nikolausz

Table S1. Operating conditions and process parameters of the eleven biogas reactors at three sampling times [for more details see Tables 1 and 2 in Ziganshin et al. (2013)]. SBP, methane content and pH values (daily measured parameters) are presented as weekly mean values (3 days before and 3 days after sampling). Standard deviations are shown for SBP (n=7).

Reactor	Sample	Substrate	OLR (g_{VS} $L^{-1}day^{-1}$)	HRT (day)	SBP (mL $g^{-1}{}_{VS}$)	Methane content	pH	VOA (g L^{-1})	Acetate (g L^{-1})	Propionate (g L^{-1})	TAN (g L^{-1})	FAN (g L^{-1})
R 3.1	1	Cattle/ chicken manures	1.78	64.4	390±8	57.5	8.04	6.09	2.44	0.60	5.17	0.754
	2		2.42	51.6	315±9	57.3	7.90	9.57	5.43	1.99	5.17	0.569
	3		2.84	45.1	290±21	57.0	7.96	15.36	9.87	4.06	5.93	0.737
R 4.5	1	Cattle manure	2.43	22.6	253±5	65.5	7.77	1.46	0.12	0.01	1.86	0.139
	2		2.45	24	348±49	63.3	7.66	1.66	nd	nd	2.01	0.126
	3		0.50	24	593±20	44.2	7.51	4.5	2.22	0.41	1.74	0.195
R 4.6	1	Cattle manure/ DDGS	2.45	24	518±22	64.5	7.63	1.46	0.04	0.01	1.65	0.091
	2		2.45	24	540±83	61.6	7.56	0.72	nd	nd	1.63	0.082
	3		0.50	24	534±92	51.7	7.21	8.78	5.58	0.64	1.62	0.096
R 4.8	1	Cattle manure/ maize silage	1.50	25	337±44	58.2	7.65	0.47	0.04	0.03	1.20	0.065
	2		1.50	25	380±46	58.5	7.35	0.14	0.03	0.001	0.94	0.026
	3		1.50	25	383±9	60.3	7.30	0.45	0.06	0.01	0.80	0.020
R 4.13	1	Cattle manure/ maize	2.47	35	400±40	58.7	7.63	1.49	0.10	0.03	1.20	0.066
	2		2.37	35	360±30	59.8	7.50	1.9	0.10	0.02	1.24	0.051

	3	straw	2.39	35	330±50	55.6	7.61	1.8	0.87	0.13	1.16	0.062
	1		2.47	35	400±30	59.3	7.66	1.42	0.07	0.01	1.22	0.072
R 4.14	2	Cattle manure/ maize straw	2.37	35	380±40	58.4	7.53	1.66	0.25	0.04	1.28	0.057
	3		2.39	35	370±50	56.7	7.58	1.43	0.45	0.04	1.31	0.065
	1		2.40	35	390±40	58.1	7.75	1.54	0.05	0.01	1.47	0.105
R 4.15	2	Cattle manure/ extruded maize straw	2.29	35	390±20	59.3	7.56	1.28	nd	nd	1.39	0.066
	3		2.30	35	390±50	56.8	7.74	1.37	0.38	0.03	1.26	0.088
	1		2.40	35	410±30	58.6	7.76	1.51	0.07	0.02	1.54	0.112
R 4.16	2	Cattle manure/ extruded maize straw	2.29	35	380±30	59.0	7.54	1.53	0.15	0.01	1.36	0.062
	3		2.30	35	390±50	57.2	7.61	1.37	0.36	0.04	1.27	0.067
	1		2.00	68.3	715±17	51.5	7.63	1.54	nd	nd	nd	nd
R 4.17	2	Cattle manure/ maize silage	2.40	41.7	588±42	52.6	7.47	6.97	nd	nd	nd	nd
	3		2.50	61.7	708±18	54.6	7.64	2.30	nd	nd	nd	nd
	1		1.84	40	491±7	64.6	7.78	2.64	nd	nd	3.22	0.245
R 4.19	2	*Jatropha* press cake	2.00	40	483±20	63.2	7.75	2.91	0.59	0.01	3.22	0.230
	3		2.20	40	475±8	62.4	7.78	3.55	1.39	0.15	3.72	0.283
	1		1.84	40	486±13	64.4	7.72	2.36	nd	nd	3.22	0.216
R 4.20	2	*Jatropha* press cake	2.00	40	464±18	64.0	7.71	3.15	0.76	0.02	3.36	0.220
	3		2.20	40	450±9	63.5	7.78	2.51	0.63	0.07	3.76	0.286

OLR – organic loading rate

HRT – hydraulic retention time

SBP – specific biogas production

VOA – volatile organic acids

TAN – total ammonium nitrogen

FAN – free ammonia nitrogen (calculated from TAN, temperature and pH according to Hansen et al. [1])

nd – not determined

Table S2. Sequencing results of representative *mcrA* gene clones and experimentally determined terminal restriction fragments (T-RF). The assignment of operational taxonomic units (OTU) is based on the highest BLASTX hit excluding environmental clone sequences. Clone designations refer to the reactor (see Table S1 for details).

Clone (bp)	Acc. No.	Highest BLASTX hit (Accession number) / Sequence identity	Taxonomic affiliation	*Hae*III T-RF (bp)	*Msp*I[b] T-RF (bp)
4.6-1-37 (429)	KX523626	*mcrA* [*Methanoculleus bourgensis*] (BAF56665) / 94%	*Methanoculleus* sp.	< 50	178
4.17-2-46 (423)	KX523627	*mcrA* [*Methanoculleus chikugoensis*] (BAF56658) / 94%	*Methanoculleus* sp.	< 50	178
**OTU 1**			***Methanoculleus* sp. I**	**< 50**	**178**
4.6-1-04 (423)	KX523628	*mcrA* [*Methanoculleus* sp. M07] (BAF46710) / 94%	*Methanoculleus* sp.	176	178
**OTU 2**			***Methanoculleus* sp. II**	**176**	**178**
3.2-3-03[a] (423)	KX523629	*mcrA* [*Methanoculleus* sp. MH98A] (WP_048114231) / 94%	*Methanoculleus* sp.	214	60
3.2-3-10[a] (423)	KX523630	*mcrA* [*Methanoculleus* sp. MH98A] (WP_048114231) / 94%	*Methanoculleus* sp.	214	60
4.17-2-13 (429)	KX523631	*mcrA* [*Methanoculleus* sp. MH98A] (WP_048114231) / 95%	*Methanoculleus* sp.	214	59
4.19-3-07 (423)	KX523632	*mcrA* [*Methanoculleus* sp. MH98A] (WP_048114231) / 95%	*Methanoculleus* sp.	214	60
4.20-2-01 (423)	KX523633	*mcrA* [*Methanoculleus* sp. MH98A] (WP_048114231) / 96%	*Methanoculleus* sp.	214	60
**OTU 3**			***Methanoculleus* sp. III**	**214**	**59/60**
3.2-3-09[a] (423)	KX523634	*mcrA* [*Methanoculleus* sp. MH98A] (WP_048114231) / 95%	*Methanoculleus* sp.	456	60
4.19-3-02 (423)	KX523635	*mcrA* [*Methanoculleus* sp. MH98A] (WP_048114231) / 96%	*Methanoculleus* sp.	457	59
4.19-3-03 (423)	KX523636	*mcrA* [*Methanoculleus* sp. MH98A] (WP_048114231) / 96%	*Methanoculleus* sp.	457	60
4.20-2-03 (429)	KX523637	*mcrA* [*Methanoculleus* sp. MH98A] (WP_048114231) / 96%	*Methanoculleus* sp.	457	60
**OTU 4**			***Methanoculleus* sp. IV**	**456/457**	**59/60**
3.2-3-01[a] (423)	KX523638	*mcrA* [*Methanoculleus bourgensis* MS2] (BAF56666) / 94%	*Methanoculleus* sp.	456	178
3.2-3-08[a] (423)	KX523639	*mcrA* [*Methanoculleus bourgensis* MS2] (BAF56666) / 100%	*Methanoculleus* sp.	456	178
4.5-3-06 (423)	KX523640	*mcrA* [*Methanoculleus palmolei*] (BAF56663) / 96%	*Methanoculleus* sp.	457	178

4.6-1-38 (429)	KX523641	*mcrA* [*Methanoculleus bourgensis*] (BAF56665) / 93%	*Methanoculleus* sp.	457	178
4.13-2-16 (429)	KX523642	*mcrA* [*Methanoculleus bourgensis*] (BAF56665) / 94%	*Methanoculleus* sp.	457	178
4.17-2-32 (423)	KX523643	*mcrA* [*Methanoculleus bourgensis*] (BAF56665) / 93%	*Methanoculleus* sp.	457	178
**OTU 5**			***Methanoculleus* sp. V**	**456/457**	**178**
4.13-2-15 (396)	KX523644	*mcrA* [*Methanocorpusculum aggregans*] (AAL29283) / 95%	*Methanocorpusculum* sp.	493	178
4.15-3-02 (424)	KX523645	*mcrA* [*Methanocorpusculum aggregans*] (AAL29283) / 99%	*Methanocorpusculum* sp.	493	178
**OTU 6**			***Methanocorpusculum* sp.**	**493**	**178**
4.20-2-06 (402)	KX523672	*mcrA* [*Methanoregula boonei*] (WP_012106121) / 83%	*Methanoregulaceae*	58	56
**OTU 7**			***Methanoregulaceae* I**	**58**	**56**
4.20-2-02 (408)	KX523673	*mcrA* [*Methanoregula boonei*] (WP_012106121) / 81%	*Methanoregulaceae*	58	91
**OTU 8**			***Methanoregulaceae* II**	**58**	**91**
4.5-3-04 (402)	KX523646	*mrtA* [*Methanobacterium formicicum*] (AAL29300) / 98%	*Methanobacterium* sp.	464	194
4.17-2-34 (402)	KX523647	*mrtA* [*Methanobacterium kanagiense*] (BAI94571) / 98%	*Methanobacterium* sp.	463	194
**OTU 9**			***Methanobacterium* sp. I**	**463/464**	**194**
3.2-3-12[a] (399)	KX523648	*mrtA* [*Methanobacterium kanagiense*] (BAI94571) / 98%	*Methanobacterium* sp.	465	465
4.6-1-34 (402)	KX523649	*mrtA* [*Methanobacterium kanagiense*] (BAI94571) / 95%	*Methanobacterium* sp.	465	465
4.17-2-12 (402)	KX523650	*mrtA* [*Methanobacterium kanagiense*] (BAI94571) / 94%	*Methanobacterium* sp.	465	465
**OTU 10**			***Methanobacterium* sp. II**	**465**	**465**
3.2-3-02[a] (402)	KX523651	*mcrA* [*Methanobacterium kanagiense*] (BAI94570) / 95%	*Methanobacteriaceae*	471	471
3.2-3-11[a] (402)	KX523652	*mcrA* [*Methanobrevibacter smithii* ATCC 35061] (ABB77886) / 96%	*Methanobacteriaceae*	471	471
4.5-3-12 (406)	KX523653	*mcrA* [*Methanobrevibacter smithii* ATCC 35061] (ABB77886) / 97%	*Methanobacteriaceae*	471	471
4.15-3-01 (404)	KX523654	*mcrA* [*Methanobrevibacter gottschalkii*] (ACK56066) / 97%	*Methanobacteriaceae*	471	471
4.17-2-16 (408)	KX523655	*mcrA* [*Methanobacterium kanagiense*] (BAI94570) / 95%	*Methanobacteriaceae*	471	471
4.19-3-01 (402)	KX523656	*mcrA* [*Methanobacterium kanagiense*] (BAI94570) / 95%	*Methanobacteriaceae*	471	471
4.19-3-10 (402)	KX523657	*mcrA* [*Methanobacterium kanagiense*] (BAI94570) / 94%	*Methanobacteriaceae*	471	471
4.20-2-04 (402)	KX523658	*mcrA* [*Methanobacterium formicicum*] (ABO93184) / 95%	*Methanobacteriaceae*	471	471

**OTU 11**			***Methanobacteriaceae***	**471**	**471**
4.6-1-02 (409)	KX523659	*mcrA* [*Methanomethylovorans thermophila*] (AAT81537) / 96%	*Methanomethylovorans* sp.	124	169
**OTU 12**			***Methanomethylovorans* sp.**	**124**	**169**
4.20-2-05 (425)	KX523660	*mcrA* [*Methanosarcina acetivorans*] (AAC43405) / 95%	*Methanosarcina* sp.	125	216
**OTU 13**			***Methanosarcina* sp. I**	**125**	**216**
4.13-2-19 (430)	KX523661	*mcrA* [*Methanosarcina thermophila* TM-1] (AAC43426) / 97%	*Methanosarcina* sp.	490	178
4.5-3-02 (426)	KX523662	*mcrA* [*Methanosarcina spelaei*] (AIF27798) / 94%	*Methanosarcina* sp.	490	178
4.5-3-05 (418)	KX523663	*mcrA* [*Methanosarcina thermophila* TM-1] (AAC43426) / 96%	*Methanosarcina* sp.	490	179
4.15-3-04 (423)	KX523664	*mcrA* [*Methanosarcina spelaei*] (AIF27798) / 94%	*Methanosarcina* sp.	490	179
4.15-3-07 (418)	KX523665	*mcrA* [*Methanosarcina thermophila* TM-1] (AAC43426) / 96%	*Methanosarcina* sp.	490	179
4.15-3-10 (418)	KX523666	*mcrA* [*Methanosarcina spelaei*] (AIF27798) / 94%	*Methanosarcina* sp.	490	179
4.15-3-11 (418)	KX523667	*mcrA* [*Methanosarcina thermophila* TM-1] (AAC43426) / 97%	*Methanosarcina* sp.	490	179
4.20-2-11 (418)	KX523668	*mcrA* [*Methanosarcina spelaei*] (AIF27798) / 94%	*Methanosarcina* sp.	490	178
**OTU 14**			***Methanosarcina* sp. II**	**490**	**178/179**
4.6-1-01 (371)	KX523669	*mcrA* [*Methanosaeta concilii* GP6] (AAK16832) / 95%	*Methanosaeta* sp.	175	193
4.6-1-05 (409)	KX523670	*mcrA* [*Methanosaeta concilii* GP6] (AAK16832) / 96%	*Methanosaeta* sp.	175	193
4.17-2-24 (405)	KX523671	*mcrA* [*Methanosaeta concilii* GP6] (AAK16832) / 96%	*Methanosaeta* sp.	175	193
**OTU 15**			***Methanosaeta* sp.**	**175**	**193**
4.17-2-36 (411)	KX523674	*mcrA* [*Methanomassiliicoccales* archaeon RumEnM2] (KQM10793) / 98%	*Methanomassiliicoccales*	198	128
**OTU 16**			***Methanomassiliicoccales***	**198**	**128**

[a] Clones originate from reactor R 3.2 which was fed with chicken and cattle manure as reactor R 3.1 (data of R 3.2 are not included into the manuscript).

[b] PCR products were not purified and due to the remaining Taq activity the clone T-RF length with *Msp*I is usually 2 bp longer than the one in the community analysis.

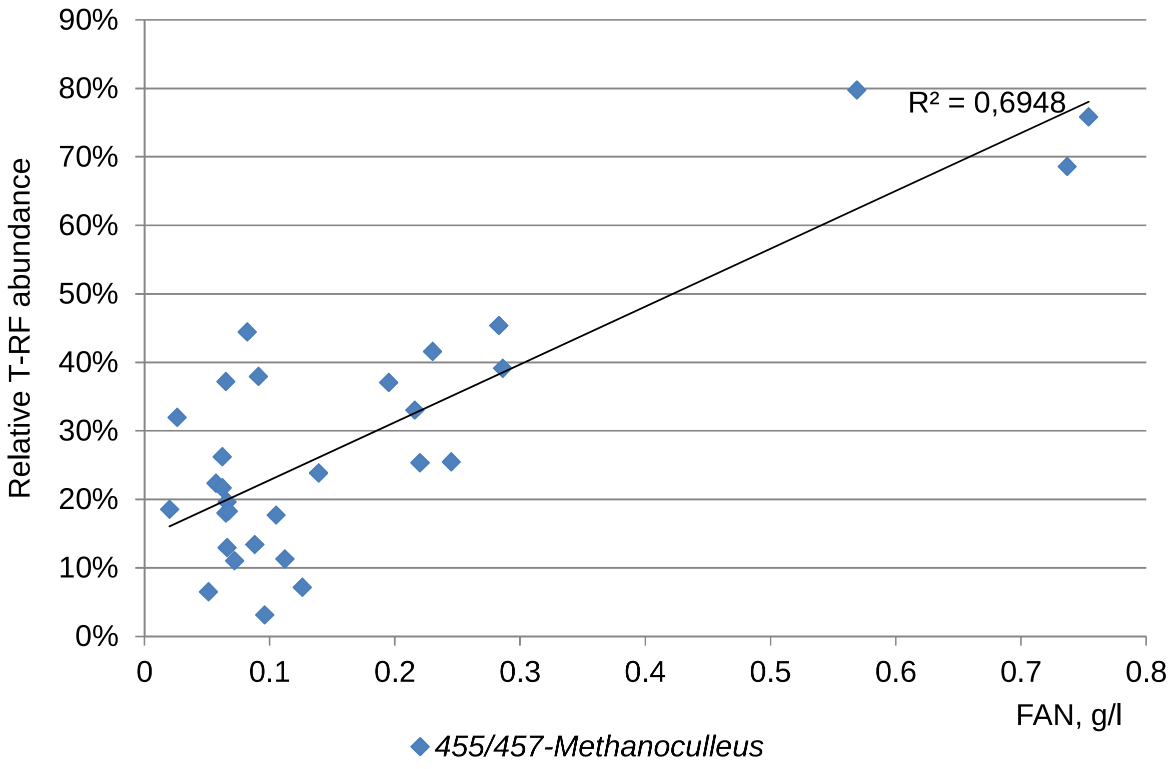

Fig. S1. Relationships between free ammonia nitrogen concentration and abundance of *Methanoculleus* with T-RF 455/457.

<u>References:</u>

[1] Hansen, K.H., Angelidaki, I., Ahring, B.K., Anaerobic digestion of swine manure: inhibition by ammonia. Water Res. vol. 32, no. 1., pp. 5–12., 1998.
